# The brain represents people as the mental states they habitually experience

**DOI:** 10.1038/s41467-019-10309-7

**Published:** 2019-05-23

**Authors:** Mark A. Thornton, Miriam E. Weaverdyck, Diana I. Tamir

**Affiliations:** 1 Department of Psychology, Princeton University, Princeton, NJ 08540 USA; 2 Princeton Neuroscience Institute, Princeton University, Princeton, NJ 08540 USA; 3 Department of Psychology, University of California, Los Angeles, Los Angeles, CA 90095 USA

**Keywords:** Human behaviour, Social neuroscience, Social behaviour, Cognitive neuroscience

## Abstract

Social life requires us to treat each person according to their unique disposition. To tailor our behavior to specific individuals, we must represent their idiosyncrasies. Here, we advance the hypothesis that our representations of other people reflect the mental states we perceive those people to habitually experience. We tested this hypothesis by measuring whether neural representations of people could be accurately reconstructed by summing state representations. Separate participants underwent functional MRI while considering famous individuals and individual mental states. Online participants rated how often each famous person experiences each state. Results supported the summed state hypothesis: frequency-weighted sums of state-specific brain activity patterns accurately reconstructed person-specific patterns. Moreover, the summed state account outperformed the established alternative—that people represent others using trait dimensions—in explaining interpersonal similarity. These findings demonstrate that the brain represents people as the sums of the mental states they experience.

## Abstract (web summary)

Social life requires us to store information about each person’s unique disposition. Here, the authors show that the brain represents people as the sums of the mental states that those people are believed to experience.

## Introduction

Humans are idiosyncratic. Two individuals placed in the same circumstances will often act in dramatically different ways. A successful social agent must recognize these idiosyncrasies, and treat each person according to their unique disposition^1,2^. For instance, a good teacher might recognize that some students are shy, and require encouragement to participate, whereas others are overconfident, and could benefit from having their assumptions challenged. How do people tailor their actions to the idiosyncrasies of specific individuals? We propose that accomplishing this task hinges upon the way people represent other people. Here, we define a person representation as the knowledge called to mind when one thinks about another person. We suggest that perceivers may construct these representations out of mental states—the thoughts and feelings—that other people habitually experience. We will refer to this idea as the “summed state” hypothesis, since it claims that people are represented as the sums of their states. This idea follows from antecedent theories in the personality^3^ and facial expression literatures^4^, which suggest traits are best measured by repeated state reports, and that face-based impression formation is based on hints of emotional expression in people’s neutral expressions.

Why might people represent other people in terms of their mental states? First, mental states are an accessible source of information about other people. Every day we observe other people experiencing a wide variety of mental states, such as joy, frustration, calm, exhaustion, decision making, or planning. As perceivers, we do not have direct access to others’ internal states. However, we can reliably infer those states by observing what people say and do, and noticing subtle cues such as tone of voice, facial expressions, and context^5,6^. Over time, we may learn that certain people experience certain states more than others^7,8^: some individuals are chronically cheerful, others habitually pensive, and so forth. Each person’s disposition and chronic environment culminate in a unique fingerprint of habitual mental states. Such individual differences in state frequencies thus provide an accessible basis for representing an individual.

Second, mental states are a useful source of information about other people. Mental states predict behavior^9,10^: angry people fight, frightened people run, ecstatic people celebrate, tired people rest, and so forth. Mental states also predict future states. By capitalizing on regularities in emotional dynamics, people can predict future emotions based on current emotions^11^. For example, if you know that someone is currently feeling awe, you can accurately predict that they are more likely to next feel joy than disgust. Thus, attending to mental states allows perceivers to gain useful glimpses of the social future.

The summed state account is not the only theory which could explain disposition-based social prediction. Traditionally, theories of person perception have assumed that people represent others using traits. Here we define traits as dimensions that capture people’s enduring dispositions. Traits differ from states in that the former rarely change, whereas the latter regularly change. If a person is “smart,” that typically means the person has enduring intelligence, whereas if a person is “ashamed,” that typically means the person is temporarily feeling shame. Certain descriptors make sense at both the trait and state level. For example, someone could be momentarily happy or a chronically happy person. That said, when we refer to traits from here out, we refer only to traditional trait dimensions, which typically cannot be reduced entirely to frequency of a certain state.

Trait theories have proven highly successful at explaining the structure of personality and social cognition^12–14^ and the neural representations of other people^2,15,16^. Moreover, like habitual mental states, traits can facilitate a wide range of social predictions. However, states offer at least two advantages over traits in this regard: (i) states are more observable than traits. States can be accurately read, or inferred in the moment^5^, but inferring traits requires disentangling complex dispositional and situational influences on behavior over time^17^. (ii) States support accurate predictions in the moment^11^, through state to state transitions. States also support representations of disposition representations through summed states. Since traits by definition do not vary over time (except, perhaps, over long periods such as years or decades) they cannot do this double-duty of supporting both momentary and habitual predictions simultaneously.

The overlap between the state and trait views of person perception has established roots in multiple domains of psychology. First, there has been a longstanding controversy in the social and personality psychology literature surrounding the validity of the trait–state distinction, with some arguing that the expression of disposition is inherently contingent on the specific situations in which people find themselves^18,19^. From this perspective, the functional difference between traits and mental states becomes small, since both constructs vary with time. That is, although traits themselves might be stable over long time periods, their expression is tied to situations which last for seconds, minutes, or hours—similar in timescale to states. Recent neuroimaging evidence also calls into question the trait–state divide, suggesting that traits and states exist in partially redundant representational spaces^15^. Experience-sampling research likewise suggests that traits reported at a single time point and averaged reports of momentary states across long periods of observation reflect a similar underlying phenomenon^20^. This finding closely approximates the current hypothesis because it sums states over time before comparing them to traits. Similar findings led to the formulation of Whole Trait Theory^3^, which suggests that traits can be better measured across repeated state reports (though cf. ref. ^21^).

Second, models of face perception suggest an intrinsic link between emotional expression and impression formation^4^. That is, faces with extreme dispositional characteristics (e.g., highly trustworthy) appear to wear emotional expressions (e.g., a smile). It has been suggested that face-based impression formation is thus driven by (potentially misleading) hints of habitual mental states in people’s faces. If so, this mechanism could explain how perceivers form impressions prior to observing affect or actions.

Finally, the summed state account accords well with the recent suggestion that mood represents emotional momentum^22^. This theory posits that moods may represent a running summary of recent reward history. The summed state hypothesis can be thought of as a natural extension of this theory: if moods cache recent momentary rewards, then dispositions may cache long-term histories of moods. This insight may allow for a closer integration of person perception with the burgeoning reinforcement learning literature. Thus, multiple lines of evidence have already converged on the idea that states and traits offer overlapping insights. Here we push this idea further to suggest that summed states are the primary proxies perceivers use to understand others’ traits.

Here we test the summed hypothesis using a combination of functional magnetic resonance imaging (fMRI), behavior, and text analysis. First, we estimate the neural representations of states, weight them by frequency, and then sum them to reconstruct activity patterns associated with specific target people. We find that summed states can accurately and specifically reconstruct person-specific activity patterns. Second, we compare the extent to which summed states and traits can explain the similarity between target people. We find that summed states explain interpersonal similarity better than other traits across all the measures we examine.

## Results

### Directly testing the summed state account

**Fig. 1:**
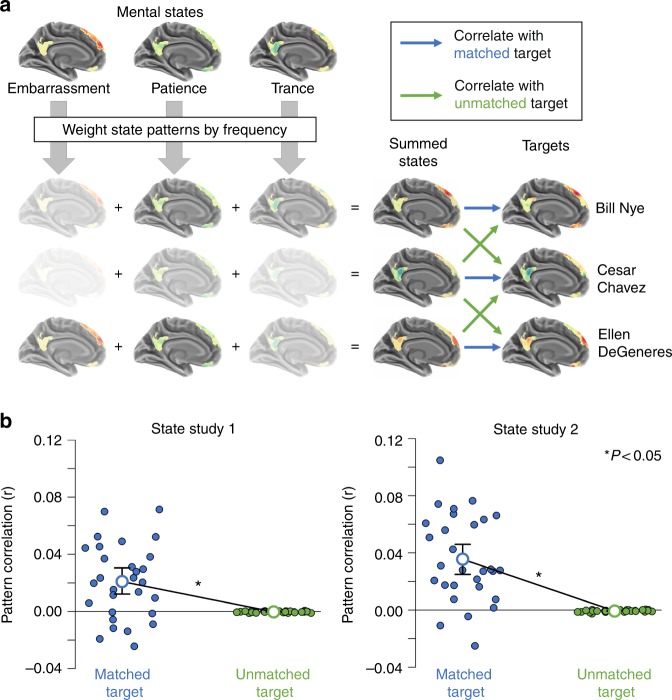
People are represented as their summed states. **a** Schematic illustration of the analysis testing the summed state hypothesis: First, estimate mental state patterns within the social brain network using fMRI in State Studies 1 and 2. Second, weight these state patterns by how frequently each target person experiences each state (via multiplication). Third, sum the weighted state patterns to reconstruct target-specific patterns. Fourth, compare the summed state patterns to target patterns, estimated in Person Study 1. Summed state patterns are compared via correlation to patterns for the correct target (matched, blue), and randomly selected targets (unmatched, green). **b** Results for State Studies 1 and 2: Summed states can accurately and specifically reconstruct the patterns representing target people. The mean correlation (open circles) between reconstructed and actual target patterns was significantly greater for matched versus unmatched targets. Error bars represent 95% bootstrap confidence intervals computed separately around each mean. The matched datapoints (blue circles) comprise the average pattern correlation across 60 actual targets for each participant in the Person Study; unmatched datapoints (green circles) comprise the average pattern correlation across 3540 unmatched targets for each participant. *P*-values reflect the results of one-sample *t*-tests across participants on differences between matched and unmatched points

The summed state hypothesis posits that the brain represents other people as the sums of their states. To test this hypothesis, we summed state-specific activity patterns from two fMRI studies—State Study 1 and State Study 2—to reconstruct target-specific activity from the Person Study (Fig. 1a). In State Studies 1 and 2, participants made judgments about sets of 60 or 15 mental states, respectively. On each trial, participants would judge which of two scenarios (e.g. “running a marathon” vs. “taking care of children all day”) would elicit more of a mental state (e.g., “exhaustion”) in another person (State Study 1), or rate how much a single scenario would elicit a given state (State Study 2). In the Person Study, participants made inferences about how well statements (e.g., “would like to learn karate” or “thinks the wealthy have a duty to the poor”) would apply to 60 famous target people (e.g., “Bill Nye”).

In all three imaging studies, we averaged over multiple presentations of the same target to estimate stable neural patterns associated with thinking about specific states and people. The state patterns were weighted by ratings of how frequently each target experiences each state, and then summed to reconstruct the target patterns. Reconstructions were compared to matched and unmatched actual target patterns to assess their accuracy, with results subjected to one-sample *t*-tests. Consistent with the summed state account, we observed that target-specific activity patterns could be accurately and specifically reconstructed by summing state representations (Fig. 1b) in both State Study 1 (mean *r* difference = 0.021, *d* = 0.81, *p* < 1.7 × 10^−4^) and State Study 2 (mean *r* difference = 0.036, *d* = 1.18, *p* < 7.6 × 10^−7^). That is, despite differences in participants, task, and scanner protocol between the Person Study and the State Studies, we nonetheless observed that weighted sums of state-specific patterns could reconstruct person-specific activity patterns. The results of this direct test of the summed state account were further supported by an indirect test conducted by applying representational similarity analysis^23^ to the same data. Mixed effects modeling of the same data likewise yields qualitatively similar results (see Supplementary Methods).

### Comparing the summed state and trait accounts

**Fig. 2:**
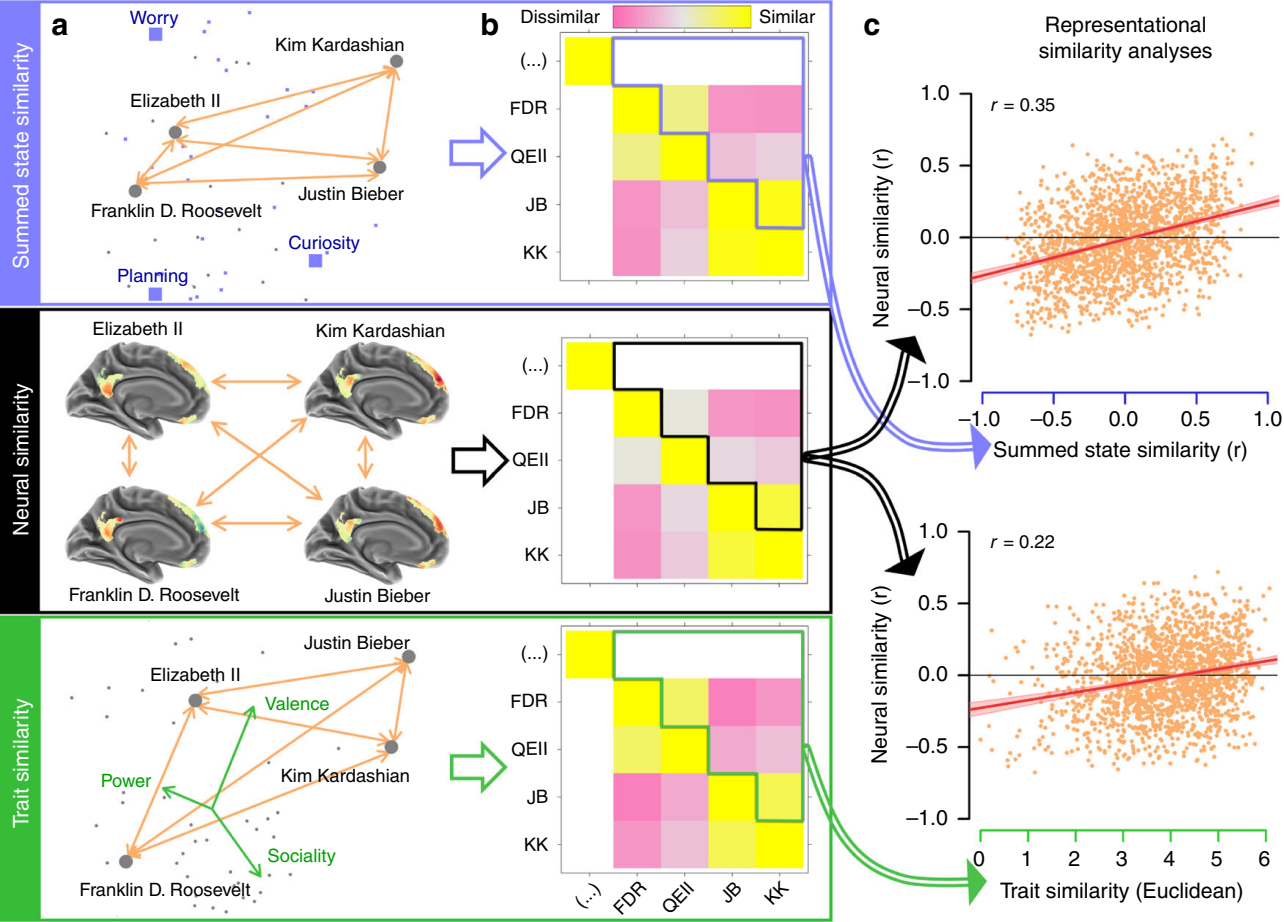
Comparing the summed state and trait accounts of person perception. **a** For each target, state frequencies (purple), neural pattern similarity (black), and trait ratings (green) were converted into the common metric of interpersonal similarity (**b**). Orange arrows indicate similarities measured in each respective space. Arrows from **a** to **b** represent the extraction of similarities from each measure. **c** The summed state similarities (top) were more strongly correlated with neural similarity than trait similarity (bottom). Arrows from **b** to **c** indicate which similarities map onto which scatter plot axes. These findings replicated using other similarity measures—ratings, choices, reaction times, and text—suggesting that summed states provide a better account of person perception. The shaded regions in the scatterplots represent 95% confidence intervals around the best fit lines

In addition to directly testing the summed state hypothesis, we also compared it to the established trait account of person perception. We examined the ability of each account to explain representations of all the target people in the Person Study. Specifically, we correlated interpersonal similarity measures with predictions generated by both the summed state account and an optimized trait account (Fig. 2). The trait account consisted of a three-dimensional synthesis (power, valence, and sociality) extracted via principal components analysis (PCA) from ratings of 13 major traits dimensions; this synthesis outperforms any individual trait theory in predicting target-specific neural activity^15^.

**Fig. 3:**
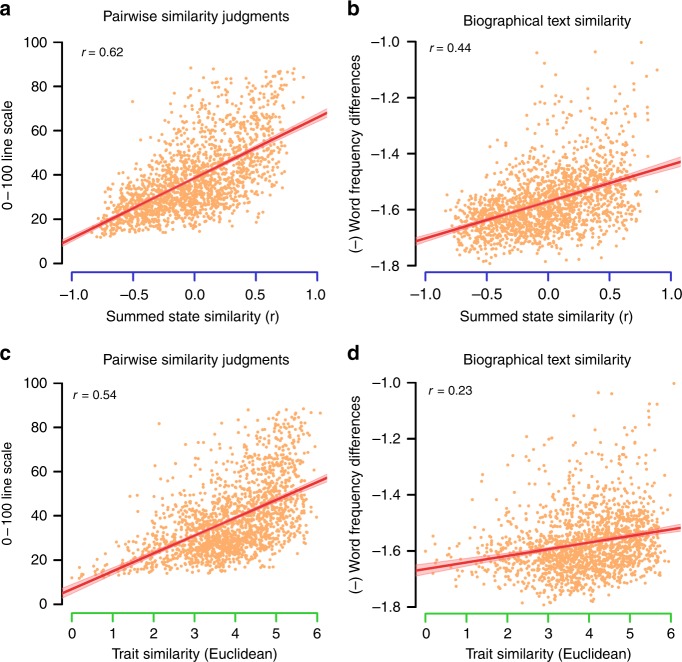
Comparing the summed state and trait accounts. **a** The correlation between summed state similarity and averaged pairwise similarity ratings. **b** The correlation between summed state similarity and the semantic similarity of biographic text. **c** The correlation between trait similarity and averaged pairwise similarity ratings. **d** The correlation between trait similarity and the semantic similarity of biographic text. Axis colors correspond to the analysis schematic (Fig. 2): summed states (blue), traits (green), and interpersonal similarity measure (black). In all cases, summed state account provides a better description of person perception than the trait account. The shaded regions in the scatterplots represent 95% confidence intervals around the best fit lines

To ensure the robustness and generalizability of our conclusions, we measured interpersonal similarity in five ways: neural patterns, pairwise similarity judgments, semantic similarity in biographic text, binary choices, and reaction times (RT; Figs. 2 and 3). Neural pattern similarity was measured as correlations between all target-specific patterns in the Person Study. Pairwise similarity judgments consisted of explicit ratings of the similarity between pairs of target people (e.g., how similar are Bill Nye and Ellen DeGeneres?). The similarity of biographical text was examined by comparing the frequencies of a broad set of content words in different target people’s Wikipedia biographies. Binary choices and reaction times were both measured in a task in which participants repeatedly judged which of two target people was more similar to a third.

The results of these comparative analyses strongly favor the summed states over traits (Table 1). For all measures, we assessed the following: (1) coefficient: how well each model accounted for interpersonal similarity, (2) semi-partial coefficient: the explanatory power of each model, controlling for the other (i.e., whether summed states fully mediated the traits or vice versa), and (3) model difference: whether one model significantly outperformed the other, after controlling each model for the other. In this vein, we also computed the Bayesian Information Criteria (BIC) associated with each model, with simple regressions replacing correlations where necessary. Depending on the type and format of the data for each measure, these questions were answered using bivariate correlations and semi-partial correlations at either the group or item level, or by using (generalized) mixed effects regressions and multiple regressions. Both the summed state and trait accounts significantly explained interpersonal similarity across all five measures. However, in each case the summed state account significantly outperformed traits, and BIC was much lower for the summed state model than the trait model. Moreover, with respect to two measures—textual similarity (Fig. 3a, c) and reaction times—the summed states fully statistically mediated the effect of traits. These results indicate that traits have no explanatory power with respect to these measures, beyond that offered by summed states. In no case was this pattern reversed: the summed state model always remained a significant predictor when controlling for traits.

Summed states predicted interpersonal similarity with large effect sizes across multiple measures. With respect to neural similarity, the group effect size of the summed state model was *d* = 2.09. Controlling for traits only slightly diminished this effect to *d* = 1.83. Using a technique for RSA correlation disattenuation^24^, we estimated that summed states accounted for 70.2% of the reliable neural variance underlying person representation at the item level. Summed states also achieved high accuracy with respect to predicting participants’ choices about interpersonal similarity: without any fitting, participants selected the option consistent with the summed state model on 67% of trials (versus 50% chance). Moreover, the superior performance of the summed state account was robust to different formulations of the trait account: qualitatively similar outcomes obtained if the three PCA-derived trait dimensions take on different weights, or if all 13 of the rated trait dimensions are used instead of the PCs (see Supplementary Methods).

**Table 1:** Comparing summed states and traits

Measure	Model	Coefficient	*p*	Semi-partial coefficient	Semi-partial *p*	Model difference *p*/CI	BIC
Neural	States	0.10	6.9 × 10^−12^	0.07	1.3 × 10^−10^	5.2 × 10^−7^	22.87
Neural	Traits	0.07	7.2 × 10^−12^	0.02	1.3 × 10^−5^		251.17
Ratings	States	0.62	2.2 × 10^−16^	0.40	2.2 × 10^−16^	2.2 × 10^−16^	13,985.15
Ratings	Traits	0.54	2.2 × 10^−16^	0.27	2.2 × 10^−16^		14,844.09
Text	States	0.44	2.2 × 10^−16^	0.33	2.2 × 10^−16^	2.2 × 10^−16^	−3209.95
Text	Traits	0.23	2.2 × 10^−16^	0.00	0.97		−2832.00
Choices	States	2.56	2.2 × 10^−16^	1.93	2.2 × 10^−16^	[0.03, 0.31]	6161.83
Choices	Traits	2.39	2.2 × 10^−16^	1.63	2.2 × 10^−16^		6276.91
RT	States	0.10	3.4 × 10^−15^	0.09	1.3 × 10^−9^	[0.02, 0.12]	12,902.22
RT	Traits	0.07	3.6 × 10^−7^	0.02	0.18		12,945.65

Neural coefficient reflects mean correlation; *p*-values from one-sample *t*-tests. Rating and text coefficients reflect item-level correlations; *p*-values from *z*-test and *t*-test. Choice coefficients reflect odds; *p*-values from *z*-tests within generalized linear mixed effects binomial models. Reaction time coefficients reflect standardized regression coefficients resulting from linear mixed effects models; *p*-values from Satterthwaite approximation. Confidence intervals (CIs) calculated by bootstrapping regressions approximating the mixed effects models. All model differences were computed based on the semi-partial coefficients and are presented on the same line as the better performing model

## Discussion

The current findings support the hypothesis that the mind and brain represent other people in terms of their habitual mental states. Specifically, we find that neural representations of people are composed of combinations of representations of the mental states those people are perceived to frequently experience. The summed state account outperformed an optimal version of the established trait account of person perception. That is, across multiple independent neural, behavioral, and text data sets, summed states provided a more robust explanation than traditional traits for how people perceive others. Together, the present findings suggest that we track other people’s idiosyncrasies using differences in the frequencies with which they experience specific thoughts and feelings.

The direct test lends strong support to the summed state hypothesis. This analysis predicted brain activity elicited by thinking about people using brain activity elicited by reasoning about mental states in different samples. None of the three imaging samples were aware of one another’s existence, nor the hypothesis that their responses would help test. Despite differences in participants, scanners, and tasks, summed states accurately reconstructed person-specific neural patterns. This finding suggests that encoding dispositions in terms of habitual mental states is spontaneous, generalizable, and reliant on a common neural code shared across brains. These reconstructions based on states are specific to individual targets, even though all target patterns were elicited with identical sets of situation items (Supplementary Table 1).

Notably, summed states were able to reconstruct person-specific patterns even when they were not particularly trait-like or long-lasting. The more trait-like a state is perceived to be, or the longer it is perceived to last, the more it contributes to pattern reconstruction accuracy (see Supplemental Methods). Interestingly, trait-diagnosticity has the opposite effect: more trait-diagnostic summed states contribute less to accurately reconstructing person-specific patterns. Importantly, even the shortest, least trait-like states (e.g., ecstasy), and minimally trait-diagnostic states could be summed to recover neural representations of people. This finding helps to further differentiate summed states from traits, by demonstrating a value of summed states that cannot be attributed to any trait-like qualities, nor to any trait impressions formed while considering a state. This latter possibility is particularly unlikely, since states elicited in the State Studies often suggested contradictory traits, if any.

Summed states consistently outperformed the trait alternative in explaining person representation, as measured using a wide variety of assessment techniques. Additional analyses indicate that this advantage cannot be attributed to differences in the number of traits versus states examined, to the equal weighting placed on each trait in the main analyses, nor to the selection of traits in this study. The traits considered were not merely a random collection, but were instead derived from the cardinal dimensions of four of the most successful modern models of person perception^13,25–29^. Although trait theories with greater explanatory power—either generally, or with respect to these particular targets—may be formulated in the future, the trait model tested here is representative of current trait-based theories of person perception, and thus provides a strong test case against which to compare summed states.

Although summed states outperform traits in the present investigation, these results do not contradict the decades of work supporting the importance of traits in person perception. In every comparative test of the summed state and trait accounts, the former performs better, but the latter is still an extremely robust predictor. That is, if one examined only the trait results in isolation, one would conclude that the present investigation provides strong support for the conclusion that people represent each other in terms of trait dimensions. However, the full set of findings suggest that summed states may subsume many of the insights offered by traits. That said, traits do retain an independent role in person perception beyond what summed states could account for, as summed states fully mediated trait prediction with respect to only a subset of the measures we examined. The present data do not offer much insight into what the residual role of traits might be. We speculate that communicating dispositional information might be one plausible possibility. Expressing a summed state representation in words could be quite tedious—“43% happy, 20% tired, 7% thoughtful, etc.” The concise nature of trait descriptors might thus be an efficient way of communicating one’s impressions of one person to another. More generally, neither summed states nor traits can be thought of as a complete theory of person representation, even in principle. For instance, it is difficult to imagine how either representational scheme could be used to encode arbitrary propositional information—such as a friend’s birthday—or episodic memories of the people we know.

The present findings also support previous research suggesting that the trait–state divide may be narrower than commonly thought^18,19^. Here we find that both traits and summed states can explain multiple aspects of person perception. Finding that trait and summed states span a similar representational space also provides convergent evidence—from the perspective of person perception—for the Whole Trait Theory of personality. This theory suggests that traits can be better measured through repeated state self-reports than a single set of trait self-reports^3,20^. Neither of these reporting measures perfectly agrees with the impressions formed by observers^21^. However, to the extent that impression formation parallels the scientific measurement of personality, the current results align with Whole Trait Theory. That is, perceivers may rely on repeatedly observing states to form impressions of other people because doing so yields more accurate representations of others’ unique dispositions. The present research focused on the content of person representation, but we look forward to future research that investigates the processes by which summed state representations might be acquired and subsequently used for prediction.

More generally, it is not clear from the present research the extent to which we should make a qualitative or quantitative distinction between summed states and traits. If traits are defined as any enduring feature of another person, then in this sense summed states are a subtype of traits, rather than an entirely distinct category. Even so, not all traits are summed states. Among the established traits we examined—drawn from some of the most influential theories of person perception—many seemed to lack a state over which to aggregate. For example, it would be odd to say that one is “feeling trustworthy”—such a statement would imply its opposite, suggesting that the speaker is not reliably trustworthy at all. Thus our comparison of summed states and traits might be viewed as a comparison of two different types of traits: summed states, and other, non-summed-state traits.

One limitation of the present work is that the target people studied here were well-known public figures. Although the sample of targets was carefully selected to cover the space of people as well as possible^15^, the criterion of fame inevitably limits this selection. The optimal trait account we consider here can accurately predict the brain activity associated with thinking about these famous people. However, previous research suggests that traits may not be particularly effective in explaining the brain activity elicited by thinking about personally familiar others; it is unclear whether the summed state account would fare any better with such targets. That said, focusing on famous targets arguably makes for a particularly hard test of the summed state account, since participants would likely have more trait than state information for famous people.

In conclusion, the present research demonstrates that people represent each other as the sums of their states. Evidence for this hypothesis comes from both direct and indirect testing with fMRI. The summed state account proved superior to the established trait account of person perception in terms of explaining multiple forms of interpersonal similarity. As such, the summed state hypothesis provides a compelling model of how the mind and brain may learn about, represent, and predict other people.

## Methods

### Participants

The current investigation draws on data from three previous studies^15,24,30^, here referred to as the Person Study, State Study 1, and State Study 2. Here we focus only on the elements of those studies germane to the present investigation. For newly collected data, we report how we determined sample size, all data exclusions, all manipulations, and all measures. All participants provided informed consent in manners approved by the Committee on the Use of Human Subjects in Research at Harvard University (Person Study and State Study 1) or the Princeton University Institutional Review Board (State Study 2 and newly collected data).

Participants in the Person Study (*n* = 29; 18 female, 11 male; age range 18–28, mean age = 22.7) and State Study 1 (*n* = 20; 16 female, 4 male; mean age = 22.7; age range = 18–27) were recruited from Harvard University study pool; participants in State Study 2 (*n* = 28; 17 female, 11 male; age range 18–22, mean age = 19.6) were recruited from the Princeton University Study pool. All imaging participants were right-handed, neurologically normal, fluent in English, and had normal or corrected-to-normal vision. In the Person Study, separate groups of online participants were recruited using Amazon Mechanical Turk to rate the pairwise similarity between target people (*n* = 648) and the positions of target people on 13 trait dimensions (*n* = 869).

Two groups of online participants provided state frequency ratings using TurkPrime^31^. One sample (*n* = 687) rated how often the 60 targets from the Person Study experienced each of the 15 states in State Study 2. Sixteen participants were excluded on the basis of being non-native English speakers or having a less-than-excellent self-reported grasp of English, and 27 for making 10 or fewer unique responses across the study, leaving a final sample of *n* = 644 (339 female, 303 male, 2 declined to indicate gender; mean age = 38.25, range 18–77). A second sample (*n* = 729) rated how often the Person Study targets experienced the other 45 states included in State Study 1 but not State Study 2. After 9 language and 11 unique response exclusions, 709 participants remained (363 female, 344 male, 1 other, and 1 declined to indicate gender; mean age = 37.11, range 17–71). Target sample size of the first group aimed to achieve a reliability of 0.90 with respect to states, based on data from previous online norming studies. The target sample size of the second group only aimed to achieve reliability of 0.80, due to the larger number of stimuli involved.

Participants completed an interpersonal similarity task on MySocialBrain.org. From an initial sample of 106 participants, 2 were excluded due to reporting technical issues during the experiment, and 1 was excluded due to responding implausibly fast (<500 ms) on more than 90% of trials, leaving a remaining sample of 103 (52 female, 45 male, 1 other, and 5 declined to indicate gender; mean age = 27.91, range 18–59). Participants were unpaid volunteers. The sample size was determined by web traffic to the website, rather than an a priori power analysis.

### Stimuli

**Fig. 4:**
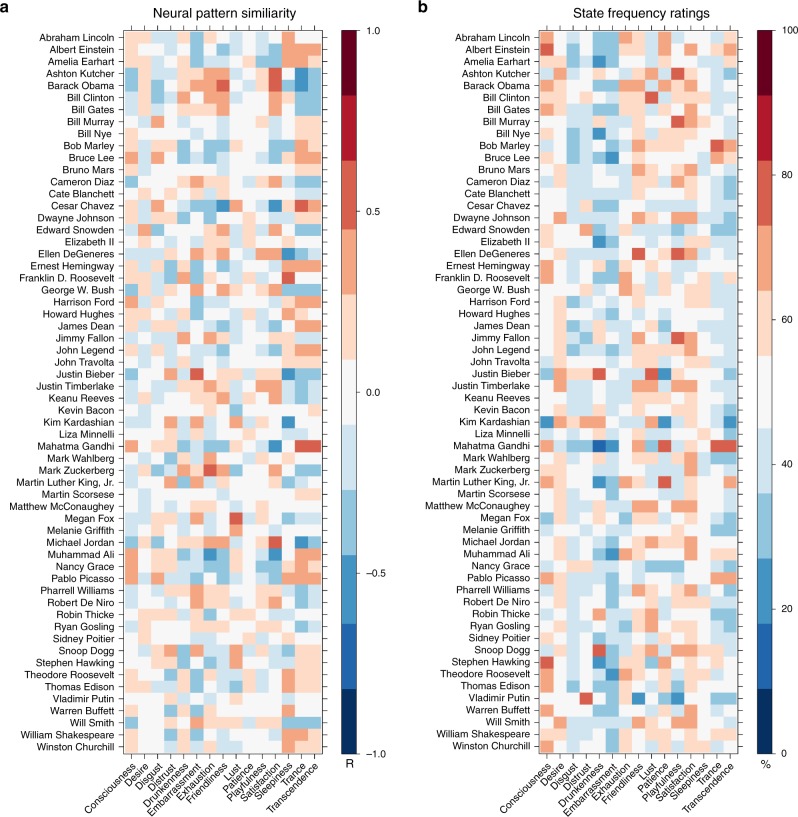
Neural pattern similarity between people and mental states, and corresponding summed states. **a** Similarity between person-specific and state-specific patterns of brain activity from two imaging experiments were calculated by correlating voxelwise values across the regions selected for containing reliable person representations. **b** Behavioral ratings of how often each person experienced each state were provided by independent online raters. These ratings were sum-normalized within state prior to analysis

The 60 famous target people (Supplementary Fig. 1 and Fig. 4) were chosen in the Person Study via a combination of text analysis and human ratings. Targets were well known, and as different from one another as possible. Biographical text similarity was measured by applying a bag-of-(content)-words approach to each target’s Wikipedia biography, and then comparing the summed absolute frequency differences. In the imaging paradigm, the target people were paired with 12 statements, such as “would like to learn karate.” These social inference statements were selected to minimize mutual redundancy using pilot data on a larger set of 24 possible statements. The selected statements (Supplementary Table 1) covered a wide space of possible social inferences, ranging from political/moral opinions “thinks the wealthy have a duty to the poor” to preferred activities “enjoys spending time in nature” to social judgments “thinks a firm handshake is important”.

In State Study 1, a representative, minimally redundant set of 60 mental states (Supplementary Fig. 1) was chosen from an exhaustive list of 166 state terms. In the imaging paradigm, these were paired with scenarios pretested to elicit the state in question (e.g., “running a marathon” for “exhaustion”). Sixteen scenarios were selected for each state from a larger set of 36 via genetic algorithm to maximize the degree to which the scenarios elicited the state in question, while maintaining balance across states. In State Study 2, a subset of 15 states (Fig. 4) were selected from the 60 used in State Study 2. This subset was chosen to maximize pairwise asymmetries in perceived state transitions^11^. Sets of 30 scenarios were selected for pairing with states in State Study 2 via genetic algorithm.

### FMRI paradigms

In the Person Study, participants underwent fMRI while thinking about famous target people. On each trial, participants made an inference about how well a statement (e.g., “would like to learn karate”) would apply to one of these targets (e.g., “Bill Nye”) using a 1–5 scale. Each trial consisted of 500 ms of target prompt, followed by a 3.25 s period to read the statement and respond, then a 250 ms fixation and a jittered fixation period (average length = 1.33 s). Across the course of 12 runs, participants rated each combination of the 60 target people and 12 statements, with both statements and targets crossed evenly with runs.

In State Study 1, participants repeatedly judged which of two scenarios (e.g. “running a marathon” vs. “taking care of children all day”) would elicit more of a mental state (e.g., “exhaustion”) in another person. Each trial consisted of a 1 s state prompt, followed by a 3.75 s period to read the scenarios and respond, then a 250 ms fixation and a jittered fixation period (average length = 1.67 s, Poisson distributed). The 60 presented mental states (Supplementary Fig. 1) were uniformly sampled from the broader population of mental state terms, with respect to the dimensions of seven existing psychological theories. Participants responded to each mental state once per run across the 16 run experiment, each time with different pairs of scenarios.

In State Study 2, participants in the imaging study repeatedly rated on a 1–5 scale the extent to which a single scenario would elicit a particular mental state. Each trial consisted of a 250 ms state prompt, followed by a 2.5 s period to read the scenario and respond, then a 250 ms fixation before the next trial. The 15 states presented in this study (Fig. 4) were a subset of those in State Study 1. Participants rated each state 15 times per run (once before each other state, including itself) in each of four runs. Scenarios were repeated twice each across the course of the experiment.

### Rating paradigms

Participants in the state frequency rating samples were directed to a Qualtrics-based web survey. In the first sample, each participant rated the extent to which each of the 60 target people would be likely to experience 1 of the 15 mental states from State Study 2. The target people were presented in a different random order for each participant. In the second sample, participants rated a subset of 100 person–state pairings from within the set of 2700 possible pairings (60 targets by 45 states in State Study 1 but not State Study 2). Ratings in both samples were made using a continuous line scale anchored at “Experiences more” and “Experiences less” of a certain state, relative to others. Participants also indicated their age, gender, and English proficiency.

### Behavioral paradigms

Participants in the interpersonal similarity study first provided their demographics: age, race, ethnicity, sex, education, and nationality. Participants then completed 50 trials of a binary choice task. On each trial, participants were shown the names of three famous target people, randomly sampled with replacement from the 60 targets in the Person Study. One of these targets would be a reference, and the other two would be choice options. Participants were instructed to indicate which of the two choices was more similar to the reference as quickly as possible. Multiple response types were allowed (clicking buttons, pressing buttons on a touchscreen device, or pressing the “1” or “2” keys on a keyboard). At the end of the study, participants indicated previous participation, technical issues, and enjoyment.

### Imaging procedure

All imaging data were collected on Siemens 3 Tesla scanners (Siemens, Erlangen, Germany)—Trio for the Person Study and State Study 1, Skyra for State Study 2—with 32-channel head coils for the Person Study and State Study 1, and a 64-channel head coil for State Study 2. Functional echo-planar BOLD images were collected with TR lengths of 2, 2.5, 1.5 s, respectively; TEs of 30, 30, and 32 ms; flip angles of 80°, 90°, and 70°; and spatial resolutions of 2, 2.5, and 2 mm isotropic voxels. All studies collected slices (69, 42, and 66, respectively) in an interleaved, axial fashion. The Person Study and State Study 2 both used simultaneous multislice acquisition, State Study 2 used parallel imaging, and State Studies 1 and 2 used prospective motion correction. High-resolution anatomical images were also acquired from participants in each study.

After data collection, imaging data were subjected to preprocessing and general linear modeling (GLM) with SPM8 (Wellcome Department of Cognitive Neurology, London, UK) with the SPM8w wrapper (https://github.com/ddwagner/SPM8w) for Person Study and State Study 1 or a multipackage pipeline (https://github.com/PrincetonUniversity/prsonpipe) combining SPM12, SPM12w, SPM8 DARTEL, and FSL^32^ for State Study 2. Preprocessing in all studies included rigid body head motion correction, normalization to ICBM 152 template (Montreal Neurological Institute) with 2 mm isotropic voxels, and 6 mm FHWM Gaussian spatial smoothing. GLMs consisted of boxcar regressors convolved with canonical hemodynamic response functions for each of the 60 targets in the Person Study, the 60 states in State Study 1, or 15 states in State Study 2. Nuisance regressors were also included to control for run means and trends, head motion, outlier time points (Person Study and State Study 1), and temporal and dispersion derivatives (State Study 1). The GLM was used to estimate stable patterns of wholebrain neural activity associated with each person or state in each dataset. That is, the GLM effectively averaged over all presentations of each target (Person Study) or each mental state (States Studies 1 and 2) to produce the pattern of activity reliably elicited in each participant’s brain by thinking about each target or state. The resulting patterns of regression coefficients for each target person/state served as the basis for the neuroimaging analysis described in this paper.

### Principal components analysis

As part of the Person Study, a group of online participants rated 60 target people on the dimensions of agency, experience, warmth, competence, trustworthiness, dominance, openness, conscientiousness, extraversion, agreeableness, neuroticism, intelligence, and attractiveness. All but the last two of these dimensions were taken from existing theories of person perception^13,25–29^. These dimensions were reduced to an optimal three-dimensional synthesis—consisting of power, valence, and sociality—via principal component analysis (PCA). The resulting synthesis outperformed any of the original trait theories of person perception in predicting patterns of brain activity^15^, and so we adopted it here for the purpose of providing the strongest trait theory against which to compare the summed state hypothesis.

### Voxel selection

**Fig. 5:**
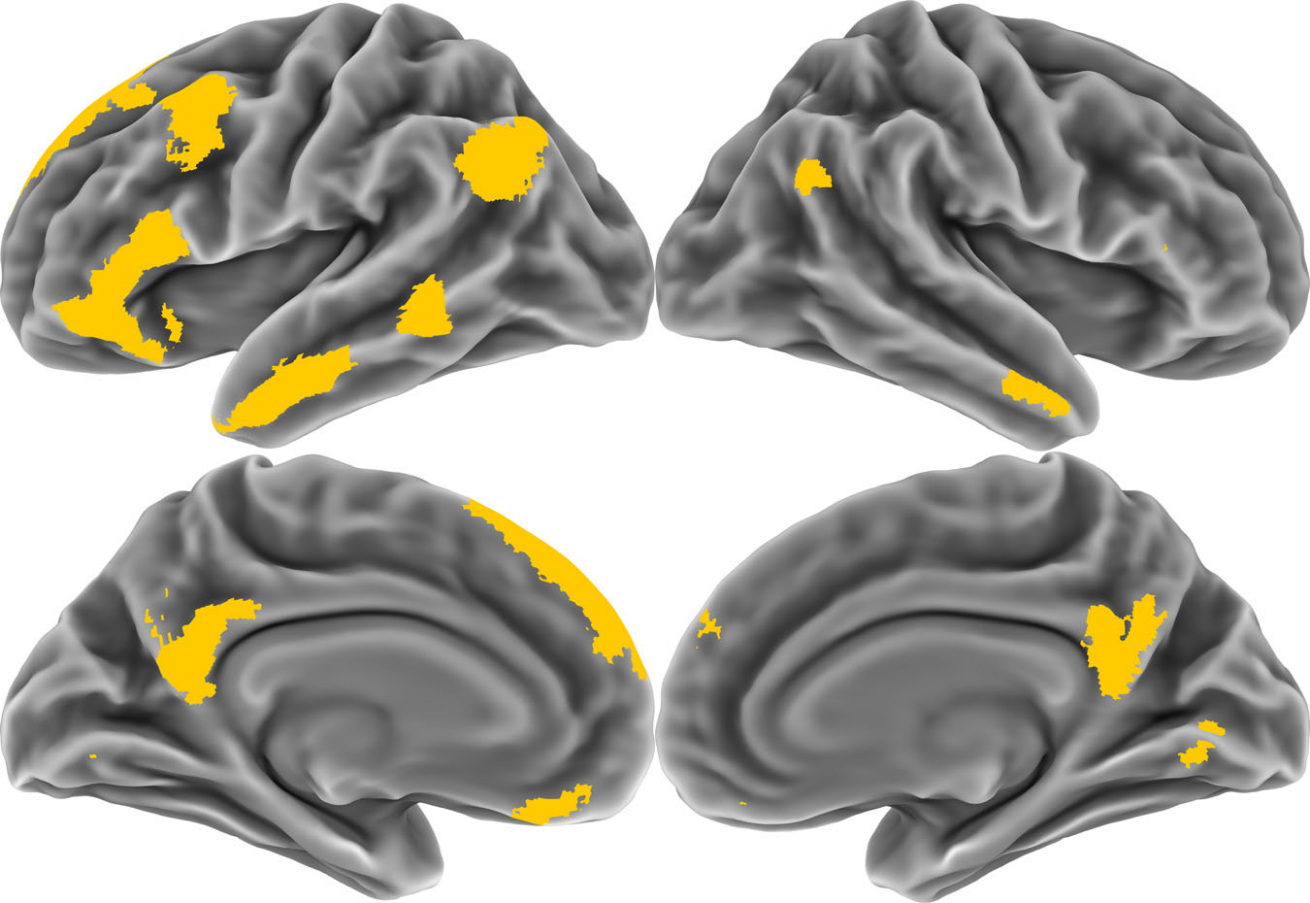
The social brain network. All fMRI analyses were conducted using patterns drawn from within the yellow regions. These voxels were selected based on the voxelwise reliability threshold which maximized the patternwise reliability of the retained voxels with respect to the target people in the Person Study

GLM-derived person-specific patterns and state-specific patterns served as the basis for all fMRI analyses. However, not all portions of the brain represent social information, and thus we performed voxel selection to limit the analyzed patterns to the social brain network. This network was defined as mask of 10,216 non-contiguous voxels implicated in social cognition^15^; these voxels maximize both voxelwise and patternwise reliability of person-related neural activity with respect to the 60 target people in the Person Study. It consisted primarily of portions of medial prefrontal and parietal cortices, the temporoparietal junction, anterior temporal lobe, and lateral prefrontal cortex (Fig. 5).

### Directly testing the summed state hypothesis

To directly test the summed state hypothesis, we attempted to reconstruct person-specific neural patterns from the Person Study with weighted averages of state-specific neural patterns from State Studies 1 and 2 (Fig. 1a), using the following steps:

1. Generate mental-state patterns: Patterns of regression coefficients were extracted from within the social brain network from the State Study GLMs. These state-specific patterns were *z*-scored within participant, and then averaged across participant, to generate a single set of state patterns for each study (60 and 15, respectively).
2. Weight state patterns by frequency: Ratings of how often each target person experienced each state were averaged across participants in the two online rating samples. This produced two rating matrices: one 60 target by 15 state matrix and one 60 target by 45 state matrix. The former of these matrices was used to weight state-specific patterns from State Study 2. The two matrices were combined to produce a 60 target by 60 state matrix for weighting state-specific patterns from State Study 1. To weight the state-specific patterns, these matrices were sum-normalized with respect first to states, and then to targets. Then each state-specific pattern was multiplied by the appropriate element of the rating matrix. For example, if the target person was “Bill Nye,” then the patience pattern would be multiplied by 0.086 (the normalized rating of how often Nye experiences patience), the embarrassment pattern would be weighted by 0.077, the trance pattern by 0.07, and so forth.
3. Sum the weighted state patterns: The weighted patterns would then be summed to reconstruct the Bill Nye pattern. This procedure yielded two sets of 60 reconstructed target patterns (one each from Studies 1 and 2).
4. Generate target patterns: Patterns of regression coefficients were extracted from the Person Study within the same set of social brain network voxels as the state-specific patterns. These target-specific patterns were *z*-scored within participant.
5. Estimate reconstruction accuracy: The accuracy of the reconstructions in each of these sets was evaluated by comparing the reconstructed patterns with the actual persons-specific pattern from the Person Study. We correlated each of the reconstructed patterns with each of the target patterns within each participant in the Person Study. This included both “matched” correlations, in which the reconstructed pattern for a given target was correlated with the actual pattern for that same target (e.g., reconstructed Bill Nye with actual Bill Nye), and “unmatched” correlations, in which a reconstructed pattern for a given target was correlated with the actual pattern for a different target (e.g., reconstructed Bill Nye with actual Ellen DeGeneres). For each matched correlation, there were 59 unmatched correlations. These unmatched correlations were averaged and then subtracted away from the corresponding matched correlation. Subtracting out the unmatched correlations eliminates the possibility that any apparent reconstruction accuracy stems from the creation of a general “person-pattern” present in every target. Any resulting accuracy must be specific to each target.

The differences between matched and unmatched correlations were then averaged within participant, and one-sample *t*-tests were used to assess statistical significance. These tests—and all statistical tests presented in this investigation—were two-sided. The entire process was repeated separately using reconstructions based on State Study 1 and State Study 2, to allow for partially independent assessments of the same hypothesis.

### Comparing summed states and traits

As a stronger test of this hypothesis, we pit summed states against the more established trait account of person perception. We compared the abilities of these two theories to predict five measures of interpersonal similarity between the target people from the Person Study.

For three of the measures considered—neural pattern similarity, pairwise similarity ratings, and the semantic similarity of biographical text—we correlated the dependent interpersonal similarity measure with predictions made by the summed state and trait accounts. Summed state predictions of interpersonal similarity were generated by correlating the combined 60 state frequency rating matrix with itself. The result was a 60-by-60 correlation matrix reflecting how similar the target people were to each other in terms of habitual mental states. Trait predictions were generated by taking the (reverse-coded) Euclidean distances between each pair of targets on the optimal trait dimensions of power, valence, and sociality. The association between state-prediction and trait-prediction, and interpersonal similarity was assessed at the item-level for all three measures, by collapsing across participants and treating elements in the similarity matrix as independent units of analysis. We repeated this procedure at the group level for the neural data, by correlating the summed state and trait predictions with the neural pattern similarity estimates of each participant in the Person Study, Fisher transforming the resulting coefficients, and then entering them into one-sample *t*-tests.

Item-level analyses were repeated using semi-partial correlations to estimate the unique explanatory power of summed states and traits when controlling for the competing account. Additionally, we directly compared the size of the correlation coefficients of the summed state and trait accounts. In the neural data, this test was conducted at the group level using one-sample *t*-tests on the differences in zero-order trait and state correlations. For the ratings and text, this comparison was performed on both the zero-order and semi-partial correlations using parametric paired correlation difference tests.

Two other measures of interpersonal similarity—binary choices and reaction times—were derived from the similarity judgment task on MySocialBrain.org. In addition to participant exclusions described above, we excluded any trial on which participants responded faster than 500 ms as implausible (2.7% of trials). We used a generalized linear mixed effects binomial regression to predict which choice (“left” or “right” target person on the screen) participants would select as more similar to the reference target. Three versions of this model were fit: one with just summed state predictions, one with just trait predictions, and one with both state and trait predictions. The predictions were derived from the trait and state similarity measures: for example, if participants were asked to indicate whether Bill Nye or Ellen DeGeneres was more similar to Cesar Chavez, then the model would predict their choice by taking the (summed state or trait) similarity between Nye and Chavez and subtracting the corresponding similarity between DeGeneres and Chavez. Random intercepts for participants were included in each model. Random slopes for the fixed effects were also included in the single-predictor models, but dropped to facilitate convergence in the two-predictor model.

We modeled reaction times using linear mixed effects models. Three versions were estimated: summed states only, traits only, and both. All three models included random intercepts for participants, and random slopes for all fixed effects models. Statistical significance was calculated using the Satterthwaite approximation for degrees of freedom. Due to the highly skewed nature of the reaction time data, we log-transformed this measure. To compare the coefficients of the state and trait predictors in the binary choice and reaction time models, we repeatedly computed general linear models (for choices) or linear regressions (for reaction times) with both summed state and trait predictors while bootstrapping with respect to participants. For each bootstrapped model, we calculated the difference between the standardized coefficients for summed states and traits. We then computed the 95% CIs for these coefficient differences. Intervals excluding zero would thus indicate a significant difference between the unique explanatory powers of summed states and traits. Note that we drop the mixed effects framework for these models for computational reasons. However, since significance estimates results from aggregation across multiple bootstrapped estimates, dropping the random effects should not impact the validity of these estimates.

### Reporting summary

Further information on research design is available in the Nature Research Reporting Summary linked to this article.

## Supplementary information

Supplementary Information

Peer Review File

Reporting Summary

## Data Availability

Data from this investigation has been deposited on the Open Science Framework (https://osf.io/gv5jm/) and are freely available.

## Code availability

Computer code used to generate and analyze the data in this investigation has been deposited on the Open Science Framework (https://osf.io/gv5jm/) and is freely available.
