# Supplementary material for: The brain represents people as the mental states they habitually experience
Source: Nat Commun. 2019 May 23;10:2291. doi: 10.1038/s41467-019-10309-7 (PMC6533269; doi:10.1038/s41467-019-10309-7)
Supplement: Supplementary file 1 — Supplementary Information [file 41467_2019_10309_MOESM1_ESM.pdf]

The brain represents people as the mental states they habitually experience

Thornton et al.

Supplementary Methods

The summed state analysis reported in the text computed the accuracy of person representation reconstruction by correlating reconstructed target patterns with matched and mismatched target patterns. For a given target, all mismatched correlations were averaged, and then these averages were subtracted from the matched correlation. The resulting differences were averaged across targets within each participant in the person study to produce that participant's reconstruction accuracy. The statistical significance of the values was assessed via a one-sample t-test against zero. There are two potential concerns with this formulation of this analysis. First, there are many more mismatched comparisons (N = 3540) than matched comparisons (N = 60). Thus, by the law of large numbers, there is much smaller variance in the mismatched means than in the matched means. This difference in variance is clearly illustrated in Figure 1B. Second, although this analysis provides random effects inference with respect to participants in the person study, it does not offer generalizability with respect to the target people themselves, because random items are not included.

To address these limitations, we repeated this analysis using a mixed effects modeling framework. We computed the accuracy of matched and mismatched pattern reconstruction in the same way as for the primary analysis. However, in the mixed effects analysis, we treated each estimate of pattern similarity as a separate observation. This obviated the need for the averaging which led to the unequal variances in the primary analysis. Linear mixed effects models were fit for each State Study. A single fixed effect term reflected whether each observation corresponded to a matched or mismatched combination of target patterns. Statistical significance of the fixed

effect was calculated using the Satterthwaite approximation for degrees of freedom. We included random intercepts for participant, reconstructed target, and actual target.

Results reaffirmed the outcome of the primary version of the summed state analysis. Matched target reconstructions were more correlated with actual target patterns for both State Study 1 (b = .021, t(104,339) = 5.69, $p < 1.3 \times 10^{-8}$) and State Study 2 (b = .036, t(104,339) = 10.22, $p < 1.6 \times 10^{-24}$). These results thus indicate that the results of the summed state analysis are not compromised by averaging artifacts, and that they can be generalized with respect to target people. Moreover, by controlling for the average reconstruction accuracy of each target person, the mixed effects modeling results also rule out the possibility that the results reflect accuracy reconstruction of any single target person.

In the present study, we contrasted an account of person representation as summed states with the established trait account of person representation. Results support the former over the latter. However, some state-descriptors are also commonly used as trait-descriptors. For example, although anyone can momentarily experience the state of being happy, it also makes sense to talk about a happy person (i.e., someone who is chronically happy). While not all state terms translate into trait terms so easily (e.g., trance, ecstasy), one might still interpret the presence of such trait-like states as further evidence for the summed state account we advance here, in that such terms are arguably explicit lexical markers of the method of summed state person representations. However, an alternative interpretation is that the summed state account only succeeds because some of the terms it considers states are actually traits. It is also possible that the tasks in the State Studies, in which participants consider the extent to which scenarios would elicit particular states, led participants to form trait impressions.

To address the latter two concerns, we collected ratings of the 60 mental states in State Study 1 on three relevant dimensions: trait-likeness (N = 52), trait diagnosticity (N = 54), and duration (N = 58). Participants were recruited from Amazon Mechanical Turk using TurkPrime[1] and forwarded to a Qualtrics-based survey. All participants provided informed consent in a manner approved by the Princeton University Institutional Review Board. Averaging ratings across participants yielded reliable composites reflecting how much each state term could be used like a trait term (trait-likeness, α = .92), how indicative each state was of people's disposition (trait-diagnosticity, α = .79), and how long each state tends to last (duration, α = .88). There were substantial positive correlations between these dimensions: r = .66 between trait-likeness and duration, r = .64 between trait-likeness and trait-diagnosticity, and r = .39 between trait-diagnosticity and duration.

If summed states accurately reconstruct neural representations of people only because some states are trait-like, trait-diagnostic, or long-lasting, then removing such states should selectively degrade the ability to reconstruct target person-specific activity patterns from state-specific activity patterns. That is, reconstructions based on states that are not trait-like, trait-diagnostic, or long-lasting should fail to accurately and specifically reflect the patterns of brain activity elicit by making social inferences about target people. To test this hypothesis, we estimated the contribution of each state-specific pattern to the accuracy of target-pattern reconstruction. We did so by repeating the primary summed state analysis reported in the text repeatedly, leaving out each mental state in turn. The principle behind this analysis was that states which make larger unique contributions to reconstruction accuracy should cause a greater drop in accuracy when removed. We focused on only State Study 1 in this instance, due to its much larger number of mental states.

For each participant in the Person Study, we regressed the results of this analysis separately onto the averaged trait-likeness, trait-diagnosticity, and duration ratings. If summed state reconstruction is enhanced by states which are trait-like, diagnostic, and long-lasting, then we should observe a negative association between each of these variables and the state-specific accuracy contribution measure we computed (given that the latter is, in effect, reverse-coded). We tested for this association via one-sample t-tests across participants in Person Study 1 on the slopes of each regression. We found that trait-likeness was indeed negatively associated with accuracy contribution (mean b = -.0009, d = .49, t(28) = -2.63, p = .014), as was state duration (mean b = -.0013, d = .63, t(28) = -3.38, p = .002). These associations indicate that trait-like and long-lasting states do contribute more to accurately reconstructing person-specific activity patterns than less trait-like and shorter duration states. Trait-diagnosticity was also significantly associated with state-specific accuracy contribution, albeit in the opposite direction (mean b = -.0026, d = 1.00, t(28) = 5.36, $p = 1.03 \times 10^{-5}$). This indicates that less trait-diagnostic states actually contribute more to summed-state representations. The reversed direction of this finding is particularly striking, given the aforementioned high positive correlations between trait-diagnosticity and the other two variables.

These results suggest that trait-likeness and state duration are significant moderators of the degree to which state frequencies contribute to neural representations of people. However, these associations do not reveal whether this moderation explains away the summed state effect, or merely qualifies it. To address this question, in the regressions above we transformed each of the rated variables so that they varied from 0 to 1. Thus, while the slopes reflected the associations between the ratings and accuracy contributions, the intercept of each regression reflected the expected reconstruction accuracy given minimal trait-likeness, diagnosticity, and

duration, respectively. As with the slopes, we tested the statistical significance of the intercepts using one-sample t-tests across participants in the Person Study. We found that these intercepts remained significantly above zero for all regressions containing trait-likeness (mean r = .019, d = .88, t(28) = 4.73, p = 5.75 x 10^{-5}), trait-diagnosticity (mean r = .017, d = .80, t(28) = 4.32, p = .0002), and duration (mean r = .019, d = .87, t(28) = 4.66, p = 6.94 x 10^{-5}). This result indicates neural representations of people can be reconstructed even by summing mental states which are not at all trait-like, not diagnostic of traits, and short in duration. That is, summed states continue to comprise representations of people, even when those states bear little resemblance to traits.

Representational similarity analysis was used to conduct an indirect test of the summed state hypothesis. The logic of this test was that, if the brain represents others as the sums of their states, then the pattern elicited by thinking about a given target person should resemble the neural patterns of the states that person habitually experiences. In this analysis, the averaged state-specific patterns from State Studies 1 and 2 were correlated with the targets-specific patterns of each participant in the Person Study. That is, a participant's Bill Nye pattern would be directly correlated with the average patience pattern, the average embarrassment pattern, the average trance pattern, and so forth. This process yielded estimates of the neural similarity between person representations and state representations (Fig. 4 A shows the average result from State Study 2).

If person representations are composed of frequency-weighted combinations of state representations, as the summed state hypothesis suggests, then we should observe that the pattern of a given target resembles the patterns of states that person experiences frequently. To test this prediction, we correlated the person-state neural similarity matrices with the corresponding state

frequency matrix (ratings of how often each target experiences each state, averaged across raters and sum normalized with respect to states; see Fig. 4 B for example from State Study 2). This process was repeated at both the item level (averaging across all participants within each study, i.e., fixed effects) and the group level (treating participants in the Person Study as the independents units of analysis). At the group level, individual correlations were linearized through Fisher's r-to-z transformation and then entered into one-sample t-tests. The entire indirect testing procedure was repeated separately for State Studies 1 and 2.

The results of this indirect test further supported the summed state hypothesis. At the item level, we observed a significant correlation between neural pattern similarity and state frequency ratings when comparing the Person Study with State Study 1 ($r = .066$, $p < 7.6 \times 10^{-5}$), or State Study 2 ($r = .19$, $p < 4.9 \times 10^{-9}$). These relationships persisted at the group level in both State Study 1 (mean $r = .022$, $d = .93$, $p < 2.8 \times 10^{-5}$) and State Study 2 (mean $r = .073$, $d = 1.35$, $p < 7.1 \times 10^{-8}$). These results indicate that the neural representations of people consistently resemble the neural representations of the mental states those people are perceived to habitually experience, thus supporting the summed state account of person representation.

The traits examined in the present data set were generated by principal component analysis (PCA) across the dimensions of four major trait theories, as well as the additional dimensions of intelligence and attractiveness. In the Person Study, this optimal 3-dimensional synthesis was found to significantly outperform the four original theories in terms of reconstructing person-specific brain activity patterns. Thus, in the primary analyses reported in the main text, we used this synthetic trait-space to provide the most compelling possible challenge to our summed state alternative. However, it is worth considering whether the use of

this model might have disadvantaged the trait account in any way. Two possibilities suggest themselves: first, the approach used in the main text assumes equal weighting on all three dimensions, which might be suboptimal, and second, the compression of multiple trait dimensions into just three components may have discarded important explanatory variance from the trait account.

To assess these two possibilities, we computed two variants of the trait representational similarity analyses. In the first, we re-weighted the trait dimensions by their eigenvalues in the PCA that produced them. Thus, components that explained more of the original trait dimensions from existing theories of person perception would receive more weight when calculating predictions about interpersonal similarity. In this variant, we found that the relationship between neural pattern similarity and trait similarity was smaller than in the primary analysis reported in the text (item analysis $r = .18$, mean $r = .064$, $d = 1.84$, $p < 1.2 \times 10^{-10}$). Similarly, the association between trait similarity and pairwise similarity judgements ($r = .50$, $p < 2.2 \times 10^{-16}$) was smaller than when calculated using the evenly-weighted components. The association between trait similarity and textual similarity was larger using this weighted variant of trait-space, but only slightly ($r = .29$, $p < 2.2 \times 10^{-16}$) and this associated remained well below the effect observed in the analogous summed state analysis ($r = .44$).

The second variant of trait-space we consider abandons the compression via PCA altogether, and instead uses all 13 rated trait-space dimensions to estimate interpersonal similarity. If PCA unfairly penalizes trait-space by discarding meaningful variance, then a variant which does not apply PCA should outperform the trait results reported in the text. On the contrary, we again observe generally worse performance with this variant relative to the 3 PC model with respect to neural pattern similarity (item analysis $r = .19$, mean $r = .062$, $d = 2.31$, p

< 6.5 x 10^{-13}), pairwise similarity judgements (r = .44, p < 2.2 x 10^{-16}), and biographical text (r = .20, p < 2.2 x 10^{-16}). This 13-dimensional variant of traits-space exhibits smaller correlations than the version reported in the text, or the first variant reported in the preceding paragraph. Together, the results of these two analysis variants suggest that the specific version of trait-space used for comparison with summed states does not influence the qualitative interpretation of the present results.

It is also worth pointing out that the difference in performance between the state and trait accounts cannot be easily ascribed to the number of traits and states considered. For example, consider the 13-trait account describe above, and a version of the summed state model based on only the 15 states from State Study 2. Even with the number of states and trait dimensions roughly equal, the state account outperforms the trait account with respect to neural pattern similarity (item analysis r = .36, mean r = .11, d = 2.00, p < 1.8 x 10^{-11}), pairwise similarity judgements (r = .63, p < 2.2 x 10^{-16}), and biographical text (r = .45, p < 2.2 x 10^{-16}). In fact, the performance of the summed state model with only 15 states is extremely similar to the performance of version with the full 60 states, suggesting that a relatively parsimonious state-space may account for perception of summed states.

To visualize the location of the target people in summed state "space", we conducted a simple correspondence analysis using the 'anacor' package [2] in R [3]. Correspondence analysis provides a way of analyzing contingency or co-occurrence tables, making it well-suited for analyzing the state-frequency ratings we collected for each target person. These ratings, averaged and normalized as described in the main text, were directly entered into the correspondence analysis, with a 2-D solution requested. The results (Figure S1) project both the mental states and

the target people into the same latent space. The proximity between states and target people reflect the state-frequency ratings, such that targets are closer to the states they are perceived to frequently experience. Proximity between different target people also reflects how similar they are to each other, in terms of summed states.

Supplementary References

1. Litman, L., Robinson, J. & Abberbock, T. TurkPrime. com: A versatile crowdsourcing data acquisition platform for the behavioral sciences. *Behavior research methods* **49**, 433-442 (2017).

2. De Leeuw, J. & Mair, P. Simple and canonical correspondence analysis using the R package anacor. *Journal of Statistical Software* **31**, 1-18 (2009).

3. R: A language and environment for statistical computing. (R Core Team, 2013).

Supplementary Figures

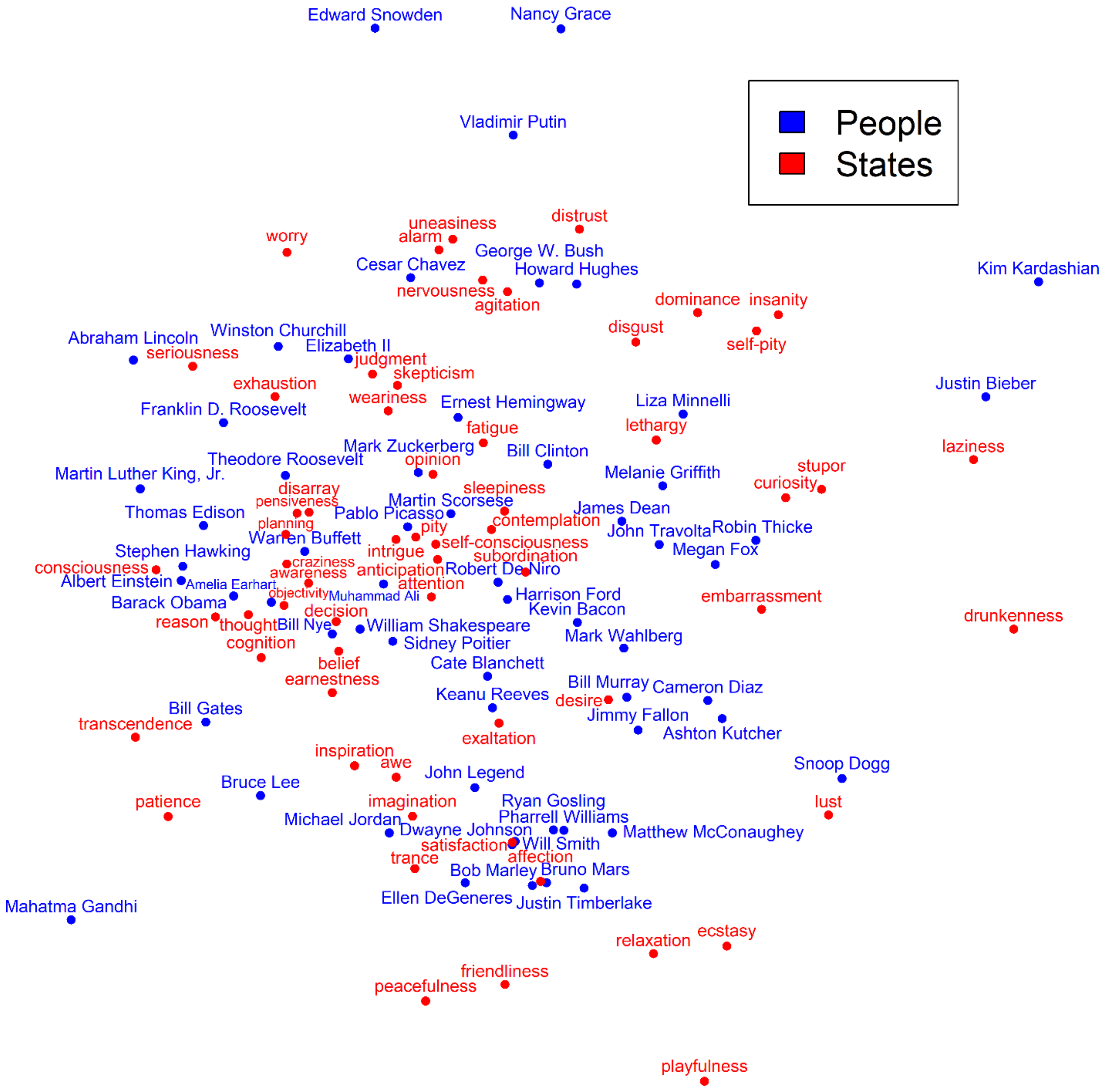

Supplementary Figure 1. *A map of summed state dispositions.* Ratings of how often each target in the Person Study was thought to experience each mental state in State Study 1 were projected into a joint space via correspondence analysis. The closer a person is to a state, the more frequently they are thought to experience it. The fact that similar people generally appear close to each other occurs only because summed states describe interpersonal similarity.

Supplementary Tables

Supplementary Table 1. Item statistics from Person Study.

Item text	Mean response	Mean *SD* of response	Mean reaction time (s)
loves to solve difficult problems	3.24	1.18	1.79
enjoys spending time in nature	3.18	0.97	1.80
enjoys learning for its own sake	3.42	1.10	1.80
likes debating issues with others	3.29	1.09	1.81
thinks that a firm handshake is important	3.41	1.00	1.82
likes to deal with problems alone	3.02	0.97	2.01
prefers to avoid conflict when possible	2.92	1.00	2.02
dislikes travelling by airplane	2.38	0.92	1.86
finds the use of profanity offensive	2.32	1.04	1.95
thinks the wealthy have a duty to the poor	3.17	1.06	1.90
would like to learn karate one day	2.89	0.99	1.83
would grieve the loss of a pet	3.51	0.78	1.75
