# Supplementary material for: The brain represents people as the mental states they habitually experience
Source: Nat Commun. 2019 May 23;10:2291. doi: 10.1038/s41467-019-10309-7 (PMC6533269; doi:10.1038/s41467-019-10309-7)
Supplement: Supplementary file 3 — Reporting Summary [file 41467_2019_10309_MOESM3_ESM.pdf]

nature research

Corresponding author(s): Mark Thornton

Last updated by author(s): Apr 20, 2019

Reporting Summary

Nature Research wishes to improve the reproducibility of the work that we publish. This form provides structure for consistency and transparency in reporting. For further information on Nature Research policies, see Authors & Referees and the Editorial Policy Checklist.

Statistics

For all statistical analyses, confirm that the following items are present in the figure legend, table legend, main text, or Methods section.

n/a	Confirmed	
☐	☒	The exact sample size (n) for each experimental group/condition, given as a discrete number and unit of measurement
☐	☒	A statement on whether measurements were taken from distinct samples or whether the same sample was measured repeatedly
☐	☒	The statistical test(s) used AND whether they are one- or two-sided<br>*Only common tests should be described solely by name; describe more complex techniques in the Methods section.*
☐	☒	A description of all covariates tested
☐	☒	A description of any assumptions or corrections, such as tests of normality and adjustment for multiple comparisons
☐	☒	A full description of the statistical parameters including central tendency (e.g. means) or other basic estimates (e.g. regression coefficient) AND variation (e.g. standard deviation) or associated estimates of uncertainty (e.g. confidence intervals)
☐	☒	For null hypothesis testing, the test statistic (e.g. F, t, r) with confidence intervals, effect sizes, degrees of freedom and P value noted<br>*Give P values as exact values whenever suitable.*
☒	☐	For Bayesian analysis, information on the choice of priors and Markov chain Monte Carlo settings
☐	☒	For hierarchical and complex designs, identification of the appropriate level for tests and full reporting of outcomes
☐	☒	Estimates of effect sizes (e.g. Cohen's d, Pearson's r), indicating how they were calculated

Our web collection on statistics for biologists contains articles on many of the points above.

Software and code

Policy information about availability of computer code

Data collection	Data in the fMRI studies were collected using PsychoPy >=1.7 in Python 2.7. Ratings of state frequencies were collected via the Qualtrics survey platform. Behavioral data in the state similarity judgment task was collected on MySocialBrain.org using Bootstrap 3.3.5, jQuery 1.11.1, and custom Javascript/CSS.
Data analysis	Imaging data were subjected to preprocessing and general linear modeling (GLM) with SPM8 (Wellcome Department of Cognitive Neurology, London, UK) with the SPM8w wrapper (https://github.com/ddwagner/SPM8w) for Person Study and State Study 1 or a multipackage pipeline (https://github.com/PrincetonUniversity/prsonpipe) combining SPM12, SPM12w, SPM8 DARTEL, and FSL for State Study 2. Neural and behavioral data were subsequently analyzed in R 3.3.

For manuscripts utilizing custom algorithms or software that are central to the research but not yet described in published literature, software must be made available to editors/reviewers. We strongly encourage code deposition in a community repository (e.g. GitHub). See the Nature Research guidelines for submitting code & software for further information.

Data

Policy information about availability of data

All manuscripts must include a data availability statement. This statement should provide the following information, where applicable:

- Accession codes, unique identifiers, or web links for publicly available datasets
- A list of figures that have associated raw data
- A description of any restrictions on data availability

Data and code have been deposited on the Open Science Framework (https://osf.io/gv5jm/) and are freely available.

Field-specific reporting

Please select the one below that is the best fit for your research. If you are not sure, read the appropriate sections before making your selection.

☐ Life sciences ☒ Behavioural & social sciences ☐ Ecological, evolutionary & environmental sciences

For a reference copy of the document with all sections, see nature.com/documents/nr-reporting-summary-flat.pdf

Behavioural & social sciences study design

All studies must disclose on these points even when the disclosure is negative.

Study description	The data from human fMRI studies were combined with large rating data sets and an online behavioral task. All studies were quantitative.
Research sample	Participants in the Person Study (N = 29; 18 female, 11 male; age range 18-28, mean age = 22.7) and State Study 1 (N = 20; 16 female, 4 male; mean age = 22.7; age range = 18-27) were recruited from Harvard University study pool; participants in State Study 2 (N = 28; 17 female, 11 male; age range 18-22, mean age = 19.6) were recruited from the Princeton University Study pool. All imaging participants were right-handed, neurologically normal, fluent in English, and had normal or corrected-to-normal vision. In the Person Study, separate groups of online participants were recruited using Amazon Mechanical Turk to rate of the pairwise similarity between target people (N = 648) the positions of target people on 13 trait dimensions (N = 869).<br><br>Two groups of online participants provided state frequency ratings using TurkPrime. One sample (N = 687) rated how often the 60 targets from the Person Study experienced each of the 15 states in State Study 2. Exclusions left a final sample of N = 644 (339 female, 303 male, 2 declined to indicate gender; mean age = 38.25, range 18-77). A second sample (N = 729) rated how often the Person Study targets experienced the other 45 states included in State Study 1 but not State Study 2. After exclusions, 709 participants remained (363 female, 344 male, 1 other, and 1 declined to indicate gender; mean age = 37.11. range 17-71).<br><br>Participants completed an interpersonal similarity task on MySocialBrain.org. After exclusion sample of 103 remained (52 female, 45 male, 1 other, and 5 declined to indicate gender; mean age = 27.91, range 18-59). Participants were unpaid volunteers.
Sampling strategy	All samples were convenience-based. This paper drew on three previously published fMRI data sets. Sample sizes in those data sets were selected based on the goals of the original studies, and were chosen via Monte Carlo simulation or resampling-based power analyses. Sample sizes for the state frequency ratings were determined to achieve preset reliability thresholds. Sample size in the experiment on MySocialBrain.org was determined by web traffic to the website.
Data collection	In the fMRI studies, the experiment was presented and data collected via computer and 3T Siemens scanner. The online studies were presented over the internet using Qualtrics or MySocialBrain.org. Only the researchers were present with the participant during the fMRI studies. We did not attempt to monitor who else might be present in the online studies. Researchers were not blinded to the experimental conditions/hypothesis.
Timing	The studies were not longitudinal in nature. Collection for various studies within the paper took place between fall 2013 and spring 2018.
Data exclusions	1 participant was excluded from the Person Study due to low response rate, and 1 participant was excluded from State Study 2 due to data loss in the image reconstruction process. In the state-frequency ratings, a total of 25 participants were excluded on the basis of language comprehension, and a further 38 were excluded for making 10 or fewer unique responses. In the online behavioral experiment 2 were excluded due to reporting technical issues during the experiment, and 1 was excluded due to responding implausibly fast (< 500 ms) on more than 90% of trials. We also excluded any remaining individual trials (2.7%) that were faster than this time limit.
Non-participation	No participants declined/withdrew from the imaging experiments. Withdrawal in the online studies was not tracked.
Randomization	All imaging studies were randomized within participant, with each participant being exposed to each condition in that study in random order. The online studies were partially crossed, in that each participant saw a random sub-sample of the total possible trials.

Reporting for specific materials, systems and methods

We require information from authors about some types of materials, experimental systems and methods used in many studies. Here, indicate whether each material, system or method listed is relevant to your study. If you are not sure if a list item applies to your research, read the appropriate section before selecting a response.

Materials & experimental systems

n/a	Involved in the study	
☒	☐	Antibodies
☒	☐	Eukaryotic cell lines
☒	☐	Palaeontology
☒	☐	Animals and other organisms
☐	☒	Human research participants
☒	☐	Clinical data

Methods

n/a	Involved in the study	
☒	☐	ChIP-seq
☒	☐	Flow cytometry
☐	☐	MRI-based neuroimaging

Human research participants

Policy information about studies involving human research participants

Population characteristics	See "research sample" section above.
Recruitment	Participants in the imaging studies were recruited from the Harvard and Princeton University study pools. Raters were recruited via Amazon Mechanical Turk. Participants on MySocialBrain.org were recruited via social media, web postings, and passive internet traffic. None of these study pools are nationally representative, but we do not anticipate any uncommon bias in the samples as a function of the content under study here.
Ethics oversight	The Committee on the Use of Humans Subjects at Harvard University and the Institutional Review Board at Princeton University approved the studies presented in the investigation.

Note that full information on the approval of the study protocol must also be provided in the manuscript.

Magnetic resonance imaging

Experimental design

Design type

Condition-rich event related designs (+ continuous carry-over in State Study 2)

Design specifications

In the Person Study, participants underwent fMRI while thinking about famous target people. On each trial, participants made an inference about how well a statement (e.g., “would like to learn karate”) would apply to one of these targets (e.g., “Bill Nye”) using a 1-5 scale. Each trial consisted of 500 ms of target prompt, followed by a 3.25 s period to read the statement and respond, then a 250 ms fixation and a jittered fixation period (average length = 1.33s). Across the course of 12 runs, participants rated each combination of the 60 target people and 12 statements, with both statements and targets crossed evenly with runs.

In State Study 1, participants repeatedly judged which of two scenarios (e.g. “running a marathon” vs. “taking care of children all day”) would elicit more of a mental state (e.g., “exhaustion”) in another person. Each trial consisted of a 1 s state prompt, followed by a 3.75 s period to read the scenarios and respond, then a 250 ms fixation and a jittered fixation period (average length = 1.67s). The 60 presented mental states (Fig. S1) were uniformly sampled from the broader population of mental state terms, with respect to the dimensions of seven existing psychological theories. Participants responded to each mental state once per run across the 16 run experiment, each time with different pairs of scenarios.

In State Study 2, participants in the imaging study repeatedly rated on a 1-5 scale the extent to which a single scenario would elicit a particular mental state. Each trial consisted of a 250 ms state prompt, followed by a 2.5 s period to read the scenario and respond, then a 250 ms fixation before the next trial. The 15 states in presented this study (Fig. 4) were a subset of those in State Study 1. Participants rated each state 15 times per run (once before each other state, including itself) in each of four runs. Scenarios were repeated twice each across the course of the experiment.

Behavioral performance measures

Button presses and reaction times were recorded in all three imaging studies. In the Person Study, ratings were subjected to mixed effects modeling to replicate previous findings using the same paradigm (see Thornton & Mitchell, 2018, Cerebral Cortex for details). In State Study 1, response rate was monitored to measure participant engagement. In State Study 2, we analyzed test-retest reliability across repeated trials to measure engagement (see Thornton, Weaverdyck, & Tamir, PsyArXiv).

Acquisition

Imaging type(s)

Functional, structural, spin-echo

Field strength

3T

Sequence & imaging parameters

For the Person Study, State Study 1, and State Study 2, functional echo-planar BOLD images were collected with TR lengths of 2 s, 2.5 s, 1.5 s, respectively; TEs of 30 ms, 30 ms, and 32 ms; flip angles of 80º, 90º, and 70º; and spatial resolutions of 2 mm, 2.5 mm, and 2 mm isotropic voxels. All studies collected slices (69, 42, and 66, respectively) in an

interleaved, axial fashion. The Person Study and State Study 2 both used simultaneous multislice acquisition, State Study 2 used parallel imaging, and State Studies 1 and 2 used prospective motion correction.

Area of acquisition	Whole brain
Diffusion MRI	☐ Used ☒ Not used

Preprocessing

Preprocessing software	Imaging data were subjected to preprocessing and general linear modeling (GLM) with SPM8 (Wellcome Department of Cognitive Neurology, London, UK) with the SPM8w wrapper (https://github.com/ddwagner/SPM8w) for Person Study and State Study 1 or a multipackage pipeline (https://github.com/PrincetonUniversity/prsonpipe) combining SPM12, SPM12w, SPM8 DARTEL, and FSL for State Study 2. Preprocessing in all studies included rigid body head motion correction, normalization to ICBM 152 template (Montreal Neurological Institute) with 2 mm isotropic voxels, and 6 mm FHWM Gaussian spatial smoothing.
Normalization	Normalization was achieved using the standard linear SPM8 algorithm in the Person Study and State Study 1. In State Study 2, the nonlinear DARTEL algorithm was used.
Normalization template	ICBM 152
Noise and artifact removal	Head motion was controlled using a combination of prospective motion correction and rigid body realignment, with the estimated parameters from the latter also included as nuisance covariates in the GLMs. Run means and trends were also included as nuisance parameters in all studies. In the Person Study and State Study 1, outlier timepoints defined by the ART toolbox were included in the regression. In State Study 1, we included temporal and dispersion derivatives in the GLM.
Volume censoring	The standard SPM wholebrain mask was applied to filter voxels outside the brain. Subsequently voxels were extracted from an ROI within the brain.

Statistical modeling & inference

Model type and settings	At the first level, fixed GLMs were applied to model activity associated with condition (target person/mental state). At the 2nd level, we conducted a novel pattern analysis which consisted of out-of-sample prediction of target-person specific patterns, based on frequency-weighted averages of state-specific activity patterns (see Fig. 1). We also conducted representational similarity analysis as both the group (summary statistic) and item analysis levels, modeling pattern similarity in terms of summed states or traits.
Effect(s) tested	In the GLM, each target person/mental state was represented by a regressor. Patterns of regression coefficients were extracted for subsequent pattern analyses by contrasting each such regressor against the mean (i.e. "1" for that regressor, "0" for all others). These patterns were then entered into the pattern analyses described above. No ANOVA/ factorial effects were tested.

Specify type of analysis: ☐ Whole brain ☒ ROI-based ☐ Both

Anatomical location(s): Social brain network, as determined via reliability-based feature selection.

Statistic type for inference (See Eklund et al. 2016)	Not applicable. Pattern analyses were conducted within pre-defined sets of voxels (ROI), so no voxelwise/clusterwise analyses were conducted.
Correction	Not applicable. No whole brain analyses were conducted, and only a single ROI was used, so it was unnecessary to control for multiple comparisons in the imaging analyses.

Models & analysis

n/a	Involved in the study
☒	☐ Functional and/or effective connectivity
☒	☐ Graph analysis
☐	☒ Multivariate modeling or predictive analysis

Multivariate modeling and predictive analysis	Patterns of brain activity were extracted from within a non-contiguous ROI sensitive to thinking about people, as generated by reliability-based feature selection. State-specific patterns were weighted by independent raters' frequency judgments, and added together to create a "summed state" representation for a particular target person. This pattern was then correlated with the actual pattern elicited by thinking about that target person in a separate study, as well as the patterns of unmatched targets. These correlations measured the accuracy of the reconstruction, and the difference between them indicated the specificity of the reconstruction to the target person in question. NHST was conducted via t-tests on the average matched-nonmatched correlation difference. In the RSAs, the person-specific patterns were correlated with each other to measure neural pattern similarity, and this similarity was then correlated with predictions generated from participants ratings of mental state frequencies or traits. PCA was used to reduce the dimensionality of the trait predictors in one version of the analysis.
