# Supplementary material for: The brain represents people as the mental states they habitually experience
Source: Nat Commun. 2019 May 23;10:2291. doi: 10.1038/s41467-019-10309-7 (PMC6533269; doi:10.1038/s41467-019-10309-7)
Supplement: Supplementary file 2 — Peer Review File [file 41467_2019_10309_MOESM2_ESM.pdf]

Editorial Note: This manuscript has been previously reviewed at another journal that is not operating a transparent peer review scheme. The manuscript was considered suitable for publication without further review at Nature Communications.
